# Exploring the Validity of Measures of Health-Related Quality of Life in Older Adults at Increased Risk of Falls and/or Fractures in Exercise Clinical Trials

**DOI:** 10.1177/07334648251316633

**Published:** 2025-02-26

**Authors:** Carrie-Anne Ng, Brendan Mulhern, Akanksha Akanksha, Mina Bahrampour, Paul Jansons, Jakub Mesinovic, Anoohya Gandham, Costas Glavas, Peter R. Ebeling, Rosalie Viney, David Scott

**Affiliations:** 1 Centre for Health Economics Research and Evaluation, 63645 University of Technology Sydney, NSW, Australia; 2 Institute for Physical Activity and Nutrition, School of Exercise and Nutrition Sciences, 110576 Deakin University, Burwood, VIC, Australia; 3 Department of Medicine, School of Clinical Sciences at Monash Health, 161662 Monash University, VIC, Australia; 4 Mary MacKillop Institute for Health Research, 625920 Australian Catholic University, VIC, Australia

**Keywords:** health-related quality of life, quality of life, psychometrics, exercise, falls, patient-reported outcome measure, EQ-5D, MFES

## Abstract

Exercise targeting physical function and body composition may mitigate falls and fracture risk among older adults. This study aimed to identify the most valid instrument(s) to assess quality of life (QoL) in this context by comparing the psychometric properties of the EQ-5D-3L, EQ-5D-5L, CDC Healthy Days measure, Modified Falls Efficacy Scale (MFES), and Work Productivity and Activity Impairment Questionnaire. Data from four exercise trials (*n* = 210, mean age 64.8 ± 7.4, 79.0% female) were analyzed. Construct validity and responsiveness were compared. There was moderate to strong convergence between the EQ-5D (-3L and -5L) and MFES, and EQ-5D-3L and CDC index (correlation: 0.45–0.61). Only the EQ-5D-3L demonstrated good known-group validity (effect size: 0.98–3.7). Responsiveness was low across all instruments (standardized response mean: −0.33–0.49). The instruments are valid for assessing QoL in older adults at risk of falls and/or fractures. However, variation in their psychometric properties should be considered when selecting instruments for exercise trials.

**What this paper adds**

• This study contributes to current evidence on the comparative psychometric performance of widely used patient-reported outcome measures (PROMs), including the EQ-5D and Modified Falls Efficacy Scale (MFES).
• The generic PROMs (EQ-5D-3L, EQ-5D-5L, and CDC Healthy Days measure) and condition-specific PROMs (MFES and Work Productivity and Activity Impairment Questionnaire (WPAI)) assessed in this study are valid instruments to assess health-related quality of life (HRQoL) in older adults at increased risk of falls and/or fractures in exercise clinical trials.
• For known-groups validity, only the EQ-5D-3L and EQ-5D-5L could discriminate between groups with higher and lower fear of falling. However, the responsiveness of all PROMs was low, indicating limited ability to detect change in HRQoL over time.

**Applications of study findings**

• Clinicians, researchers, and policy-makers should be aware of the varying levels of evidence for different psychometric properties of PROMs when assessing HRQoL at the dimension level.
• This study highlights possible areas where PROMs or new instruments could improve the measurement of HRQoL in exercise trials targeting musculoskeletal health improvements in older adults.

## Introduction

Population ageing contributes to the increasing burden of falls and fractures globally (Tatangelo et al., 2019; Lee et al., 2018). Exercise interventions targeting improvements in physical function and body composition have been shown to reduce the rate of fall-related fractures in older adults by 26% (relative risk: 0.74, 95% confidence interval: 0.59–0.92) (Wang et al., 2020) and may thus alleviate this health burden (Zhao, Feng, & Wang, 2017). A common approach to evaluating the cost-effectiveness of such interventions is through generic preference-based measures (Brazier, Ratcliffe, Saloman, & et al., 2017). These measures are typically HRQoL questionnaires, with two elements—a “descriptive system” which includes a set of items that individuals complete to describe their own health and a utility “value set” which includes a score for each health state described (anchored on a scale from 0 (death) to 1 (full health)). The resulting values are used as the “quality” element of the quality-adjusted life years used in cost-utility analyses. One such preference-based measure is the EQ-5D, which has been widely used in population studies of chronic diseases, including osteoporosis and obesity, and clinical trials of exercise training in older adults (van Dongen, Haveman-Nies, Doets, Dorhout, & de Groot, 2020; Marshall-McKenna et al., 2021). However, the psychometric performance of the EQ-5D to assess HRQoL in the setting of exercise interventions among older adults is unclear, resulting in potentially inaccurate quality-adjusted life year estimates (Davis, Liu-Ambrose, Khan, Robertson, & Marra, 2012; Taylor Susan Lord Kathryn, 2001).

Condition-specific PROMs developed to assess HRQoL are also available, and due to their content may capture different aspects of a health condition. For example, the Quality of Life Questionnaire of the European Foundation for Osteoporosis (QUALEFFO-41) has commonly been used in studies of older adults with osteoporosis (Gao & Zhao, 2023). However, there currently is limited evidence for the QUALEFFO-41’s responsiveness and its application in exercise interventions (Choo, Mohd Tahir, Mohamed, & et al., 2024).

Fear of falling is associated with HRQoL in older adults, as well as incident falls and fractures (Schoene et al., 2019; Pua, Ong, Clark, Matcher, & Lim, 2017). A systematic review identified the MFES to have moderate- to high-quality psychometric evidence for assessing falls efficacy, defined as an individual’s confidence and belief in their ability to perform daily activities without falling (Soh, Lane, Xu, Gleeson, & Tan, 2021). However, the ability of the MFES to detect change in falls efficacy over time, particularly in longitudinal studies or interventions where change is anticipated (i.e., its responsiveness), is unclear (Dabkowski, Missen, Duncan, & Cooper, 2023), although evidence suggests it may be more sensitive than generic PROMs (Hand et al., 2022). Alternative PROMs to measure HRQoL in older adults can also be considered, such as those extensively used in population health surveillance (e.g., Center for Disease Control and Prevention (CDC) Healthy Days measures (Slabaugh et al., 2016)) or measures of work productivity and activity impairment (e.g., the WPAI (Grimani, Aboagye, & Kwak, 2019)).

Further work is needed to establish the validity of PROMs to inform clinical research and practice, and economic decision-making. This study aimed to explore and compare the psychometric performance of preference-based (EQ-5D-3L and EQ-5D-5L) and profile (CDC Healthy Days, MFES, and WPAI) instruments used in exercise interventions targeting improvements in physical function and body composition in older adults at increased risk of falls and/or fractures. We hypothesized that the instruments would demonstrate convergent validity and that the EQ-5D would have similar psychometric properties to the profile instruments with regard to known-group validity and responsiveness.

## Methods

### Data Sources

Data were obtained from four existing clinical trials of exercise which included the EQ-5D (-3L or -5L) and one or more condition specific measures (including the MFES, CDC Healthy Days, or WPAI). All studies were pilot trials investigating the feasibility and safety of resistance and/or moderate-to-high impact weight-bearing exercise aimed at improving physical function and body composition to reduce falls and/or fracture risk in Australian older adults.

Details of the interventions, inclusion criteria, and PROMs administration time-points are described in Supplemental Table 1 (Mesinovic et al., 2023; Ng, McMillan, Humbert, Ebeling, & Scott, 2021; Gandham et al., 2023). Datasets A and C comprised older adults who were overweight or obese (*n* = 110), and Datasets B and D comprised postmenopausal women with low bone mass (*n* = 100). Datasets A and B prescribed exercise training to all participants, Dataset C compared gym-based and home-based aerobic exercises, and Dataset D compared home-based exercises delivered by voice-controlled personal assistants with general education.

No identifiable data were available for any dataset. The Monash Health (HREC/15/MonH/182, HREC/16/MonH/364, HREC/18/MonH/399) and Deakin University (DUHREC 2021-008) Human Research and Ethics Committees approved these studies. Written informed consent was obtained from all participants.

### Measures

#### EQ-5D

The EQ-5D is a widely used generic preference-based instrument that measures health status across five dimensions (mobility, self-care, usual activities, pain/discomfort, and anxiety/depression). The original version of the EQ-5D (EQ-5D-3L) uses three response options for each dimension (no, some, or extreme problems) (Rabin & de Charro, 2001), while the newer version (EQ-5D-5L) expands the range of responses to five levels (no, slight, moderate, severe, or extreme problems) (Herdman et al., 2011). The EQ-5D-3L was administered in Datasets A and B, and the EQ-5D-5L was administered in Datasets C and D. The Australia-specific EQ-5D (-3L and -5L) value sets were used to obtain utility values (Norman et al., 2023; Viney et al., 2014).

#### Center for Disease Control and Prevention (CDC) Healthy Days

The CDC Healthy Days Core Module is a generic four-item instrument that has been widely used in assessing and monitoring population health internationally (Moriarty, Zack, & Kobau, 2003; Duncan et al., 2014). Item 1 assesses self-rated general health with five responses ranging from excellent to poor. Items 2 and 3 assess the number of days in the past 30 days of impaired physical or mental health, respectively, and item 4 assesses the number of days in the past 30 days of limitations in usual activities due to poor physical or mental health. The summary index of unhealthy days is calculated by summing the responses to the physically unhealthy and mentally unhealthy days (items 2 and 3). If the sum is greater than 30, a maximum score of 30 is assigned. This summary index of unhealthy days assumes a minimal logical overlap of reported physically and mentally unhealthy days (Centers for Disease Control Prevention, 2001). An unhealthy days index of 14 or more was used to indicate frequent physical and mental distress as it corresponds to the upper 10%–15% distribution for each of the CDC Healthy Days indices (Brown et al., 2003).

#### Modified Falls Efficacy Scale (MFES)

The MFES is a 14-item self-report scale measuring confidence in one’s ability to perform activities of daily living without falling. The MFES is an expanded version of the original 10-item activity Falls Efficacy Scale by including four additional items on outdoor activities (Hill, Schwarz, Kalogeropoulos, & Gibson, 1996). Each item is scored on a 10-point visual analogue scale: 0 = not confident or not sure at all; 5 = fairly confident or fairly sure; and 10 = completely confident or completely sure. The total score is an average of all 14-item scores, with higher scores reflecting more confidence and less fear of falling. A score below the previously published normative value of 9.8 was used to denote higher fear of falling (Hill, Schwarz, Flicker, & Carroll, 1999).

#### Work Productivity and Activity Impairment Questionnaire (WPAI)

The WPAI has been broadly used in clinical trials of exercise to assess work productivity (both paid and unpaid work) and regular activity impairment over the past 7 days (Grimani et al., 2019). It consists of 6 items about: employment status; hours missed due to health problems; hours missed due to other reasons; hours actually worked; the degree health problems affected productivity while working (from 0: no effect to 10: complete impairment); and the degree health problems affected regular activities (from 0 to 10). Four scores are obtained: absenteeism (work time missed); presenteeism (impairment while at work); overall work productivity (absenteeism + presenteeism); and daily activity impairments. Scores are expressed as percentages, with higher scores indicating greater productivity loss (Reilly, Zbrozek, & Dukes, 1993).

### Data Analysis

#### Construct Validity

The assessment of construct validity involves evaluating how well a measure reflects the hypothesized HRQoL within a population, considering there is presently no consensus on the most valid instrument to measure HRQoL in the context of exercise interventions in older adults. This lack of a gold standard is partly due to the heterogeneity of chronic conditions among older adults, and the challenge of creating an indicator that fully assesses the impact of increased falls and fracture risk on quality of life. To assess construct validity, two related empirical tests of (a) convergent validity and (b) known-group validity were conducted on each dataset.

##### Convergent Validity

Convergence between the PROMs was tested using Spearman’s correlation coefficients. A correlation of 0.1–0.29 is considered weak, 0.3–0.49 moderate, and ≥0.5 strong (Cohen, 1992). Hypothesized item correlations were based on the similarity of item content and set a priori where at least a moderate correlation was expected between corresponding items. Agreement between the instruments was examined using Bland–Altman plots, which visually represents the relationship between two quantitative measures. Half widths of the 95% limits of agreement were calculated using 1.96 standard deviations to define the limits within which 95% of the differences should lie (Giavarina, 2015). An even distribution of points above and below the mean of the two measurements would indicate no systematic bias of one measure compared to the other.

##### Known-Group Validity

Known-group validity was assessed by testing whether each PROM summary score discriminated between different known groups. The following known groups were used: higher and lower fear of falling based on the MFES cut-off value of 9.8 (Hill et al., 1999); frequent distress based on CDC unhealthy days index cut-off value of 14 (Brown et al., 2003); the presence or absence of obesity, defined as a body mass index of ≥30 kg/m^2^; and the presence or absence of osteoporosis, defined as a bone mineral density T-score of ≤−2.5 (as assessed by dual-energy x-ray absorptiometry) (Kanis, 1994). These groups were selected as they are anticipated to score differently from others, allowing us to examine whether the PROMs are sensitive to these differences.

The magnitude of differences in the scores between groups was determined using one-way ANOVA. Standardized effect sizes across subgroups were calculated as the difference in mean scores between two adjacent severity subgroups divided by the standard deviation of scores for the milder subgroup. Effect sizes of 0.2–0.49 were considered small, 0.5–0.79 moderate, and ≥0.8 large (Cohen, 1992), with a larger effect size signifying better discriminating ability.

#### Responsiveness

To measure responsiveness, we examined ceiling and floor effects at the instrument and item level. Ceiling or floor effects were considered to be present if >15% of respondents achieved the lowest or highest possible score, respectively (Terwee et al., 2007). The magnitude of change in scores before and after the intervention was assessed using the standardized response mean (SRM) statistic (calculated by dividing the mean change on the measure by the standard deviation of the change). SRMs of 0.2–0.49 are considered small, 0.5–0.79 moderate, and ≥0.8 large (Cohen, 1992).

Meaningful change in HRQoL can be assessed using anchor-based methods, which include comparing changes in HRQoL scores to clinically relevant measures such as performance-based tests or response to intervention. The stair climb power test (SCPT) is a measure of lower extremity power that has been validated among community dwelling older adults (Ni, Brown, Lawler, & Bean, 2017). The SCPT was conducted at baseline and the final follow-up appointment in Datasets A, B, and C. Participants were instructed to safely ascend a 10-step flight of stairs (1.75 m in vertical height) as fast as possible. Timing for the test began on the investigator’s cue and stopped when both of the participants’ feet reached the top step. The average time (s) of the two trials was taken, and power (W) was calculated.

Change in SCPT was used as an anchor for responsiveness. Participants were categorized as having “improvement,” “no change,” or “deterioration” according to increases greater than, within the range of, or decreases greater than previously published minimal detectable change of 45.6 W, respectively (Ni et al., 2017). Responsiveness was tested for the overall sample, for exercise and control groups, and for groups with improvement, no change, or deterioration in SCPT.

## Results

Baseline characteristics of participants in each dataset are reported in Supplemental Table 3. At baseline, Dataset C had the highest EQ-5D and MFES scores, and lowest CDC unhealthy days index, indicating better quality of life and lower fear of falling (Table 1). Across the datasets, completion rates were generally high (>95%) except for the MFES, which was lowest among the PROMs in Datasets A, C, and D. The MFES items with the lowest completion rates were regarding “using front or rear steps at home” (94.8%) and “using public transport” (95.2%).

**Table 1.** Descriptive Statistics of PROMs Utility and Summary Scores and Completion Rates.

	Number of participants who completed the full measure	Mean	SD	Completion %
EQ-5D-3L				
Dataset A (*N* = 50)				
Baseline	50	0.851	0.169	100.0
12 weeks	42	0.862	0.230	
24 weeks	39	0.862	0.222	
Dataset B (*N* = 50)				
Baseline	48	0.903	0.122	96.0
16 weeks	44	0.885	0.136	
EQ-5D-5L				
Dataset C (*N* = 60)				
Baseline	58	0.930	0.054	96.7
12 weeks	48	0.929	0.058	
Dataset D (*N* = 50)				
Baseline	50	0.913	0.096	100.0
6 months	48	0.916	0.097	
12 months	43	0.896	0.147	
MFES				
Dataset A (*N* = 50)				
Baseline	47	9.605	0.820	94.0
12 weeks	43	9.751	0.512	
24 weeks	39	9.820	0.348	
Dataset B (*N* = 50)				
Baseline	49	9.721	0.595	98.0
16 weeks	41	9.853	0.375	
Dataset C (*N* = 60)				
Baseline	55	9.740	0.407	91.7
12 weeks	45	9.721	0.369	
Dataset D (*N* = 50)				
Baseline	43	9.553	1.260	86.0
6 months	46	9.627	1.236	
12 months	35	9.753	0.582	
CDC unhealthy days				
Dataset A (*N* = 50)				
Baseline	49	5.367	8.679	98.0
12 weeks	43	4.198	5.941	
24 weeks	39	7.756	9.403	
Dataset B (*N* = 50)				
Baseline	48	6.427	9.649	96.0
16 weeks	43	8.535	11.016	
Dataset C (*N* = 60)				
Baseline	57	4.965	8.487	95.0
12 weeks	46	5.522	8.500	
WPAI (overall work productivity loss)				
Dataset D (*N* = 50)				
Baseline	15	13.470	25.470	88.2
6 months	13	13.850	28.440	
12 months	14	12.790	26.820	

Abbreviations: SD, standard deviation; MFES, Modified Falls Efficacy Scale; CDC, Center for Disease Control and Prevention; WPAI, Work Productivity and Activity Impairment Questionnaire.

In Dataset D, 34% (*N* = 17) indicated that they were currently employed, but 12% of these participants provided invalid responses for items 2 to 4 of the WPAI (i.e., stating 0 hours) and thus could not be assigned a summary score.

### Convergent Validity

Across all datasets, there were moderate to strong correlations between the EQ-5D (both -3L and -5L) and MFES (Table 2). The EQ-5D mobility item was moderately to strongly correlated with the MFES items for walking around the house, crossing roads, and using steps at home in most datasets, which supports our hypotheses (Supplemental Table 4). Dataset D had the strongest convergence for all hypothesized item correlations between the EQ-5D and MFES (r = −0.42 to −0.79).

**Table 2.** Convergent Validity of PROMs Utility and Summary Scores at Baseline.

	EQ-5D-3L	EQ-5D-5L	MFES
Dataset A			
MFES	0.61*		-
CDC unhealthy days	−0.54*		−0.26
Dataset B			
MFES	0.45*		-
CDC unhealthy days	−0.45*		−0.28
Dataset C			
MFES		0.52*	-
CDC unhealthy days		−0.01	0.11
Dataset D			
MFES		0.74*	-
WPAI			
Overall work productivity loss		−0.81*	−0.71*
Activity impairment due to health		−0.78*	−0.74*

Spearman’s correlation coefficients are reported.

*p < .05.

Abbreviations: MFES, Modified Falls Efficacy Scale; CDC, Center for Disease Control and Prevention; WPAI, Work Productivity and Activity Impairment Questionnaire.

The EQ-5D-3L and CDC unhealthy days index were moderately correlated. In line with our hypotheses, there were moderate to strong correlations between the EQ-5D-3L usual activities and the CDC limitations to usual activities items, and between the EQ-5D-3L anxiety/depression and CDC impaired mental health items. However, the EQ-5D-3L mobility item was weakly correlated with the CDC impaired physical health item (r = 0.05–0.12). Additionally, correlations between the EQ-5D-5L and the CDC at both the instrument and item levels indicated weak convergence.

WPAI summary scores were strongly correlated with the EQ-5D-5L and MFES. The EQ-5D-5L usual activities item was also strongly correlated with the WPAI items for health problems affecting work productivity and regular activities, which supported our hypotheses.

**Figure 1.**
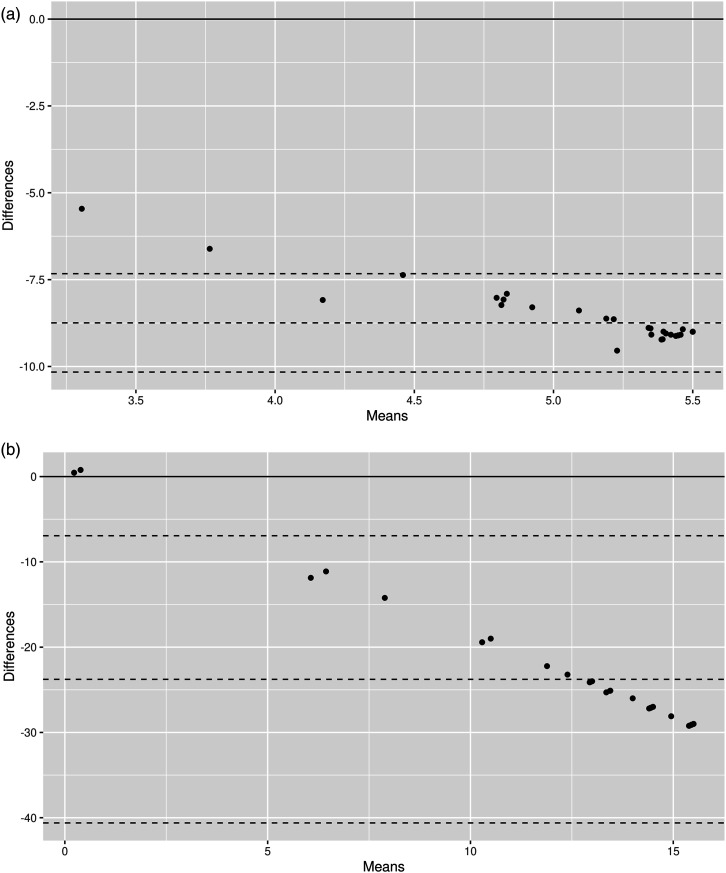
Bland–Altman plots of differences for utility and summary scores between (a) EQ-5D-3L and MFES and (b) EQ-5D-3L and CDC unhealthy days index in Dataset A (*n* = 50).

Figure 1 illustrates Bland–Altman plots comparing scores of the EQ-5D-3L and that of the MFES and CDC unhealthy days index, respectively, in Dataset A. Mean differences were −8.74 and −23.78, and limits of agreement were −7.33 to −10.2 and −6.93 to −40.61, respectively. Both plots show rather equal distribution of points above and below the line of mean difference, suggesting there was no systematic bias of one instrument over the other. However, differences in scores between instruments tend to decrease as the values increase, indicating proportional bias. Distributions were similar in the other datasets (Supplemental Figure 1).

### Known-Group Validity

EQ-5D (-3L and -5L) index scores were significantly greater in the group with lower fear of falling than those with higher fear of falling across all datasets, with large effect sizes (1.1–3.7) (Supplemental Table 5). Conversely, the CDC unhealthy days index and WPAI overall work productivity loss were not significantly different between groups with higher and lower fear of falling and demonstrated very small effect sizes (<0.15). EQ-5D-3L index scores, but not those of the EQ-5D-5L or MFES, were significantly greater in the group without frequent distress than with distress in the Datasets A and B, with large effect sizes (0.98–2.25).

All summary scores were not significantly different in the groups with osteoporosis or obesity compared with the groups without, with effect sizes in the small to moderate range (0.09–0.54). Only the MFES demonstrated a large effect size of 1.03 between the groups with and without osteoporosis, but this was not statistically significant.

### Responsiveness

At baseline, there were substantial ceiling effects (>15%) for summary indices of all instruments, indicating no problems, no fear of falling, zero unhealthy days, or no work productivity loss (Table 3). Only the exercise arm and SCPT improvement subgroup of Dataset C did not have ceiling effects for the EQ-5D-5L and moderate SRMs for EQ-5D-5L index scores. Moderate SRMs for EQ-5D-3L and MFES total score were also observed in the deterioration subgroup of Dataset A.

**Table 3.** Responsiveness of PROMs (i.e., Ability to Detect Change Over Time) for the Overall Dataset and Subgroups Based on Improvement or Deterioration in Physical Function.

	% at floor		% at ceiling^a^				
	Baseline	Follow-up	Baseline	Follow-up	Mean change (SD)	SRM	*p*-value
EQ-5D-3L							
Dataset A							
Overall (*n* = 39)	0	2.6	30.8	43.6	−0.01 (0.18)	−0.08	0.64
Improvement^b^	0	0	50.0	50.0	0.00 (0.12)	0.00	0.99
No change	0	3.6	25.0	42.9	0.00 (0.18)	0.02	0.92
Deterioration	0	0	50.0	50.0	−0.16 (0.27)	−0.57	0.34
Dataset B							
Overall (*n* = 43)	0	0	51.2	46.5	−0.02 (0.10)	−0.19	0.22
Improvement	0	0	36.4	45.5	−0.01 (0.10)	−0.14	0.66
No change	0	0	54.8	45.2	−0.02 (0.10)	−0.21	0.25
Deterioration	0	0	100	100	NA	-	-
EQ-5D-5L							
Dataset C							
EX (*n* = 24)	0	0	4.2	8.3	0.02 (0.05)	0.42	0.03*
CON (*n* = 24)	0	0	20.8	12.5	−0.02 (0.09)	−0.23	0.13
Improvement	0	0	12.5	12.5	0.03 (0.05)	0.65	0.11
No change	0	0	9.1	9.1	0.00 (0.08)	−0.03	0.89
Deterioration	0	0	20.0	20.0	0.02 (0.09)	0.20	0.68
Dataset D							
EX (*n* = 23)	0	0	17.4	21.7	−0.03 (0.14)	−0.21	0.32
CON (*n* = 20)	0	0	15.0	20.0	0.00 (0.11)	0.03	0.84
MFES total score							
Dataset A							
Overall (*n* = 39)	0	0	66.7	61.5	0.04 (0.39)	0.10	0.54
Improvement	0	0	83.3	66.7	−0.06 (0.15)	−0.40	0.37
No change	0	0	60.7	57.1	0.11 (0.35)	0.32	0.10
Deterioration	0	0	100	75.0	−0.36 (0.71)	−0.50	0.39
Dataset B							
Overall (*n* = 40)	0	0	67.5	70.0	0.08 (0.44)	0.19	0.31
Improvement	0	0	70.0	80.0	0.09 (0.22)	0.38	0.26
No change	0	0	69.0	69.0	0.09 (0.50)	0.17	0.36
Deterioration	0	0	0	0	NA	-	-
Dataset C							
EX (*n* = 23)	0	0	34.8	26.1	0.01 (0.55)	0.02	0.67
CON (*n* = 22)	0	0	40.9	40.9	0.00 (0.28)	0.01	0.99
Improvement	0	0	28.6	0	−0.21 (0.20)	−1.09	0.03*
No change	0	0	30.0	45.0	0.24 (0.37)	0.64	0.01*
Deterioration	0	0	40.0	40.0	−0.22 (0.57)	−0.39	0.43
Dataset D							
EX (*n* = 16)	0	0	68.8	75.0	0.12 (0.39)	0.30	0.35
CON (*n* = 15)	0	0	73.3	66.7	−0.08 (0.24)	−0.33	0.17
CDC unhealthy days index							
Dataset A							
Overall (*n* = 39)	2.6	7.7	46.2	35.9	4.17 (8.46)	0.49	0.04*
Improvement	0	0	50.0	50.0	3.33 (8.36)	0.40	0.37
No change	3.6	3.6	50	39.3	6.20 (9.13)	0.39	0.05*
Deterioration	0	50.0	0	0	2.75 (7.76)	0.86	0.18
Dataset B							
Overall (*n* = 41)	7.3	9.8	51.2	46.3	1.67 (7.85)	0.21	0.23
Improvement	10.0	0	40.0	40.0	−0.05 (8.94)	−0.01	0.99
No change	6.7	13.3	56.7	46.7	2.50 (7.51)	0.33	0.08
Deterioration	0	0	0	100	NA	-	-
Dataset C							
EX (*n* = 24)	8.3	4.2	54.2	45.8	2.38 (7.56)	0.31	0.17
CON (*n* = 21)	4.8	9.5	52.4	28.6	1.38 (12.83)	0.11	0.91
Improvement	0	12.5	75.0	25.0	6.75 (8.83)	0.76	0.07
No change	0	0	47.4	47.4	0.42 (5.37)	0.08	0.74
Deterioration	0	0	20.0	0	−9.00 (8.49)	−1.06	0.08
WPAI (overall work productivity loss)							
Dataset D							
EX (*n* = 7)	0	0	57.1	57.1	−4.29 (35.99)	−0.12	0.76
ON (*n* = 7)	0	0	10	100	0 (0.00)	-	-

*p < .05.

^a^Ceiling effects reported in this table are the highest possible scores reflecting better quality of life or more favorable outcomes.

^b^Improvement, no change, and deterioration subgroups were according to changes in stair climb power test between baseline and follow-up. Abbreviations: SD, standard deviation; SRM, standardized response mean; EX, exercise; CON, control; MFES, Modified Falls Efficacy Scale; CDC, Center for Disease Control and Prevention; WPAI, Work Productivity and Activity Impairment Questionnaire.

Across the other subgroups, SRMs for the MFES total score and most of the MFES items were small (Supplemental Table 6). There was no evidence of responsiveness for MFES items regarding “preparing a simple meal” and “reaching into cabinets and closets” in any dataset. However, there were moderate to large SRMs in the unexpected direction for MFES total score and CDC unhealthy days index in the improvement subgroup of Dataset C.

The CDC unhealthy days index significantly increased with moderate responsiveness only in Dataset A and associated deterioration subgroup. Although SRMs for the physically unhealthy days and activity limitation items indicated small to moderate increases in Datasets A and C, these were accompanied by a small improvement in self-reported health. The WPAI summary score remained largely unchanged in Dataset D. However, SRMs were small to moderate for most WPAI items.

## Discussion

Four datasets were used to assess the psychometric properties of four instruments—the EQ-5D (-3L and -5L), CDC Healthy Days measure, MFES, and WPAI—in pilot exercise clinical trials that aimed to reduce falls and fracture risk in older adults. Our results suggest that these generic and condition-specific PROMs were valid for use in older adults with low bone mass or obesity. Our hypothesis that the PROMs had similar psychometric performance was largely supported, except for the EQ-5D-3L, which demonstrated better discriminatory ability for groups with lower and higher fear of falling, and groups with and without frequent physical and mental distress. However, across all instruments, there was limited evidence for distinguishing the presence and absence of HRQoL impacts of osteoporosis or obesity, and responsiveness was generally low.

Our hypotheses for construct validity were largely supported, but there were notable discrepancies in correlations at the item level. Although the self-care domain of the EQ-5D assesses “problems washing or dressing” oneself, the MFES item assessing confidence to “take a bath or shower” was more strongly correlated with the self-care domain than the item “get undressed and dressed.” It is possible that issues with washing oneself may be more influential on the self-care domain or that the items are perceived and completed differently by older adults (i.e., the severity of the problem does not necessarily reflect one’s confidence in performing it) (Lay et al., 2023). Likewise, the MFES items assessing confidence in “crossing roads” and “using front or rear steps at home” were more strongly correlated with the EQ-5D mobility domain than the MFES item “walk around the inside of your house.” Indeed, the mobility domain may more likely be interpreted by older adults as allowing them the independence to perform day-to-day tasks outside the home (Keeley, Al-Janabi, Lorgelly, & Coast, 2013).

We observed weak to moderate convergence between the EQ-5D (-3L and -5L) and CDC unhealthy days index, consistent with a previous comparison in a large population-based survey (Derkach, Al Sayah, Ohinmaa, Svenson, & Johnson, 2022). The study similarly reported a high proportion of older adults indicating problems in the mobility and pain/discomfort dimensions despite reporting zero unhealthy days. Based on our item-level results, it is possible that the CDC unhealthy days index may not capture impacts on physical health as effectively as it does for mental health. Furthermore, we found that convergent validity was stronger between the CDC unhealthy days index and EQ-5D-3L, compared to that of the EQ-5D-5L. The EQ-5D-3L also outperformed the EQ-5D-5L when discriminating between groups with or without frequent physical and mental distress, despite having greater ceiling effects. It is possible that the added levels of the EQ-5D-5L were limited in improving sensitivity and that reporting “slight problems” did not greatly impact one’s evaluation of days when their mental or physical health was not good (Konnopka & Koenig, 2017).

Several differences in instrument content may explain the differences in psychometric properties between PROMs in this study. Firstly, recall period varies between all instruments, with “today” for the EQ-5D, “over the past seven days” for the WPAI, “the past 30 days” for the CDC Healthy Days, and no recall period for the MFES. Recent studies have reported that worse HRQoL tends to be reported when asked to recall the last week compared to the last day (Peasgood, Caruana, & Mukuria, 2023). While longer recall periods of greater than 2 weeks may be more appropriate for chronic conditions, a suitable recall period would also depend on the duration of the clinical trial and frequency of clinic visits (Norquist, Girman, Fehnel, DeMuro-Mercon, & Santanello, 2012). Secondly, the frequency-response format of the CDC Healthy Days and WPAI may have affected participant’s interpretation of items. Cognitive interviews with older adults with osteoporosis suggest that a severity-response format of questions, like that of the EQ-5D and MFES, is more acceptable and comprehensible than frequency responses (Naegeli, Nixon, Burge, Gold, & Silverman, 2014). Thirdly, despite the routine use of the MFES, content validity has only been adequately assessed in a study conducted in 2002 (Soh et al., 2021; Karström, Yttergren, Borgblad, & et al., 2002). Several items may now be irrelevant, including “answer the door or telephone” and “using public transport,” given the ubiquitous use of smartphones and declining engagement with public transport in suburban locations (Eady & Burtt, 2019). Weak convergence for these MFES items with the EQ-5D was observed, suggesting that further evaluation of the MFES is required. Lastly, the CDC Healthy Days measure was developed in the U.S. (Moriarty et al., 2003), and cross-cultural validation in an Australian population, to determine if it performs as well as it does in the U.S. context, has not yet been conducted.

The findings of this study highlight the limitations to sensitivity and responsiveness of PROMs in exercise trials, and have implications for the design of future trials. These inconsistencies can be consequential when comparing outcomes with other interventions and allocating resources when there is no conclusive evidence for the most valid PROMs to use. Although we observed that none of the PROMs had adequate responsiveness in all three change subgroups, there were significant improvements in clinical outcome assessments of physical function and body composition in the Datasets A and B, and mean adherence of the interventions ranged between 85% and 97% (Mesinovic et al., 2023; Ng et al., 2021). Substantial ceiling effects for all the PROMs’ summary scores were likely to impact the instruments’ ability to detect meaningful changes. Some of the constructs measured across the exercise interventions, such as work productivity and mental health, may also have been too “distal” in nature—that is, they were more likely to be affected by factors beyond the intervention—and were therefore less likely to be responsive than “proximal” concepts, like physical function (Wilson & Cleary, 1995). Physical function–specific instruments that are valid for use in older adults, such as the Patient-Reported Outcomes Measurement Information System (PROMIS) physical function scale, PROMIS physical function computer adaptive test (CAT), and SF-36 physical function sub-scale (SF-36-PFS), may thus offer greater sensitivity and content validity (Morgan, Kallen, Okike, Lee, & Vrahas, 2015; Houck, Jacobson, Bass, Dasilva, & Baumhauer, 2020; Bohannon & DePasquale, 2010), and should be considered for inclusion and further psychometric evaluation in future exercise clinical trials. The limitations of generic and condition-specific PROMs to evaluate exercise interventions also raise the possibility of developing a measure to reflect concepts that matter the most to older adults with osteoporosis or obesity. Such an instrument could be based on existing conceptual frameworks, such as the International Classification of Functioning (ICF) framework for osteoporosis, which includes one’s ability to work or engage in exercise, and maintenance of independence (Ziebart, Page, & MacDermid, 2020). Indeed, older adults have broader perceptions of quality of life that go beyond the health statuses narrowly defined by commonly used generic PROMs (Milte et al., 2014). There has been growing interest in capability instruments, such as the ICEpop CAPability measure for Older people (ICECAP-O) and the Adult Social Care Outcomes Toolkit (ASCOT) (Hackert, Exel, & Brouwer, 2017), which may supplement PROMs in exercise interventions, conducted in particular settings (e.g., ASCOT in a social care context). Overall, the choice of PROM(s) in a clinical trial should be made based on the intended purpose of the PROM and outcomes that are relevant to both the intervention and population of interest. Using a range of measures (e.g., both generic and condition-specific PROMs), while taking into account respondent burden, is thus likely to capture various constructs of interest.

## Limitations

This study’s findings should be considered alongside its limitations. The datasets used were obtained from pilot studies with heterogenous participant characteristics, exercise interventions, and goals, which could have impacted HRQoL differentially. Due to their differences, we did not pool the data in this study. Additionally, sample sizes were considered small according to PROM selection guidelines (Mokkink et al., 2018). Nonetheless, the evidence presented in this study may be useful in future systematic reviews, where psychometric results could be pooled across studies to draw conclusions about the measure properties of PROMs (Mokkink et al., 2018). We also did not analyze responsiveness against an anchor for Dataset D, as there were no valid patient-reported or clinical anchors collected. As the PROMs evaluated constructs other than physical functioning, it is possible that they detected discrepancies in emotional functioning or the presence of other comorbidities (e.g., mental health conditions). However, this was difficult to ascertain as indicators of other health conditions were not available.

## Conclusion

This study contributes valuable evidence about the psychometric performance of PROMs used in clinical trials of exercise training aiming to reduce falls and fracture risk in older adults. The EQ-5D, MFES, CDC Healthy Days measure, and WPAI are valid measures in this population. However, there are varying levels of evidence for different psychometric properties of each instrument, which clinicians, researchers, and policy-makers should be aware of. Our study also highlights possible areas where alternative PROMs or new instruments could improve the measurement of HRQoL among older adults at increased risk of falls and/or fractures.

## Supplemental Material

Supplemental Material - Exploring the Validity of Measures of Health-Related Quality of Life in Older Adults at Increased Risk of Falls And/Or Fractures in Exercise Clinical Trials Supplemental Material for Exploring the Validity of Measures of Health-Related Quality of Life in Older Adults at Increased Risk of Falls And/Or Fractures in Exercise Clinical Trials by Carrie-Anne Ng, Brendan Mulhern, Akanksha Akanksha, Mina Bahrampour, Paul Jansons, Jakub Mesinovic, Anoohya Gandham, Costas Glavas, Peter R. Ebeling, Rosalie Viney, and David Scott in Journal of Applied Gerontology
