# Supplementary material for: Exploring the Validity of Measures of Health-Related Quality of Life in Older Adults at Increased Risk of Falls and/or Fractures in Exercise Clinical Trials
Source: J Appl Gerontol. 2025 Feb 26;44(10):1711–23. doi: 10.1177/07334648251316633 (PMC12420939; doi:10.1177/07334648251316633)

Supplementary Table 1 Summary of datasets and measures used for analysis

Dataset	Description of study	Time points of measures	Preference-based measure	Profile measure	Sample size (baseline)
**D-Ex Study; Dataset A** (2016-2020)<br>Vitamin D Supplementation and Exercise for Improving Physical Function in Overweight and Obese Older Adults With Low Vitamin D Levels	RCT of vitamin D supplementation plus exercise, or placebo plus exercise over 24 weeks. Exercise is progressive resistance and aerobic training with supervised- and home-based components.<br><br>Sample includes older adults (aged 50-80 years) with BMI of 25-40 kg/m^2 and low vitamin D status (serum 25OHD ≤49.9nmol/L).	Baseline<br>12 weeks<br>24 weeks	EQ-5D-3L	CDC Healthy Days;<br>MFES	Total:<br>N=50
**Osteo-Dev; Dataset B** (2018-2019)<br>Wearable Devices for Assessing Exercise Targeting Bone Health in Post-Menopausal Women with Low Bone Mineral Density	Single arm study of home-based progressive unilateral hopping over 16 weeks.<br><br>Sample includes postmenopausal women with low bone mass (BMD T-score <1.0).	Baseline<br>16 weeks	EQ-5D-3L	CDC Healthy Days;<br>MFES	Total:<br>N=50
**OSMOSIS-P; Dataset C** (2019-2021)<br>Osteogenic Exercise for Musculoskeletal and Metabolic Health during Weight Loss in Sarcopenic Obese Older Adults: A Pilot Study	RCT of gym-based high intensity resistance and impact training plus dietary weight-loss, or home-based aerobic exercise plus dietary weight-loss over 12 weeks.<br><br>Sample includes older adults (aged 60-89 years) with BMI ≥28 kg/m^2, body fat percentage ≥30 (men) or ≥40 (women), and mobility limitation (SPPB score ≤ 11 out of 12).	Baseline<br>12 weeks	EQ-5D-5L	CDC Healthy Days;<br>MFES	Gym-based:<br>N=30<br><br>Home-based:<br>N=30
**INTERPRETER-OS; Dataset D** (2022)<br>A Pilot Feasibility Trial of Voice-Controlled Intelligent Personal Assistants as a Telehealth Self-Management Tool for Postmenopausal Women with Osteoporosis	RCT of VIPA device providing home-based exercise program and nutritional advice, or general educational material on managing osteoporosis over 6 months.<br><br>Sample includes postmenopausal women with osteoporosis.	Baseline<br>6 months<br>12 months	EQ-5D-5L	MFES;<br>WPAI	VIPA Intervention:<br>N=25<br><br>General education:<br>N=25

Abbreviations: RCT, randomised controlled trial; BMI, body mass index; 25OHD, 25-hydroxyvitamin D; CDC, Center for Disease Control and Prevention; MFES, modified falls efficacy scale; BMD, bone mineral density; HADS, hospital anxiety and depression scale; SPPB, short physical performance battery; VIPA, voice-controlled intelligent personal assistant; WPAI, Work Productivity and Activity Impairment Questionnaire.

Supplementary Table 2 Items hypothesised to be correlated (indicated by green cells) between PROMs

<table>
<tr><th rowspan="2"></th><th colspan="5">EQ-5D</th></tr>
<tr><th>Mobility</th><th>Self-care</th><th>Usual Activities</th><th>Pain/ Discomfort</th><th>Anxiety/ Depression</th></tr>
<tr><td colspan="6">MFES</td></tr>
<tr><td>Dressed/undressed</td><td></td><td style="background:#70AD47"></td><td></td><td></td><td></td></tr>
<tr><td>Simple meal</td><td></td><td></td><td></td><td></td><td></td></tr>
<tr><td>Bath/shower</td><td></td><td style="background:#70AD47"></td><td></td><td></td><td></td></tr>
<tr><td>In/out of chair</td><td></td><td></td><td></td><td></td><td></td></tr>
<tr><td>In/out of bed</td><td></td><td></td><td></td><td></td><td></td></tr>
<tr><td>Answer door/telephone</td><td></td><td></td><td></td><td></td><td></td></tr>
<tr><td>Walk around house</td><td style="background:#70AD47"></td><td></td><td></td><td></td><td></td></tr>
<tr><td>Reach into cabinet/closet</td><td></td><td></td><td></td><td></td><td></td></tr>
<tr><td>Light housekeeping</td><td></td><td></td><td style="background:#70AD47"></td><td></td><td></td></tr>
<tr><td>Simple shopping</td><td></td><td></td><td></td><td></td><td></td></tr>
<tr><td>Public transport</td><td></td><td></td><td></td><td></td><td></td></tr>
<tr><td>Crossing roads</td><td style="background:#70AD47"></td><td></td><td></td><td></td><td></td></tr>
<tr><td>Light gardening/washing</td><td></td><td></td><td style="background:#70AD47"></td><td></td><td></td></tr>
<tr><td>Using steps at home</td><td style="background:#70AD47"></td><td></td><td></td><td></td><td></td></tr>
<tr><td colspan="6">CDC</td></tr>
<tr><td>Self-reported health</td><td></td><td></td><td></td><td></td><td></td></tr>
<tr><td>Days physical health not good</td><td style="background:#70AD47"></td><td></td><td></td><td></td><td></td></tr>
<tr><td>Days mental health not good</td><td></td><td></td><td></td><td></td><td style="background:#70AD47"></td></tr>
<tr><td>Limitations in usual activities</td><td></td><td style="background:#70AD47"></td><td style="background:#70AD47"></td><td></td><td></td></tr>
<tr><td colspan="6">WPAI</td></tr>
<tr><td>Hours of work missed due to health</td><td></td><td></td><td></td><td></td><td></td></tr>
<tr><td>Hours of work missed due to other</td><td></td><td></td><td></td><td></td><td></td></tr>
<tr><td>Hours actually worked</td><td></td><td></td><td></td><td></td><td></td></tr>
<tr><td>Health affect work productivity</td><td></td><td></td><td style="background:#70AD47"></td><td></td><td></td></tr>
<tr><td>Health affect daily activities</td><td></td><td></td><td style="background:#70AD47"></td><td></td><td></td></tr>
</table>

Supplementary Table 3 Baseline characteristics for each dataset

	Dataset A			Dataset B	Dataset C			Dataset D			
	**Overall (n=50)**	Vitamin D (n=26)	Placebo (n=24)	**Overall (n=50)**	**Overall (n=60)**	Gym-based (n=30)	Home-based (n=30)	**Overall**	Intervention (n=25)	Control (n=25)	*p-value*[a]
**Age** (years; mean (s.d.))	**59.8 (6.4)**	59.6 (6.7)	60.0 (6.2)	**64.4 (7.7)**	**69.6 (6.0)**	69.5 (6.6)	69.7 (5.5)	**64.3 (6.1)**	63.5 (6.1)	65.1 (6.1)	<0.001
**Female**, %	**62.0**	61.5	62.5	**100.0**	**58.3**	63.3	53.3	**100.0**	100.0	100.0	<0.001
**Highest level of education,** %											<0.001
Primary school	**0**	0	0	**0**	**0**	0	0	**0**	0	0	
Secondary school	**16.3**	19.2	13.0	**24.0**	**19.3**	20.7	17.9	**12.0**	8.0	16.0	
Technical or similar	**18.4**	7.7	30.4	**16.0**	**19.3**	20.7	17.9	**26.0**	16.0	36.0	
University or higher	**65.3**	73.1	56.5	**60.0**	**61.4**	58.6	64.3	**62.0**	76.0	48.0	
**Marital status,** %											0.014
Single	**10.4**	15.4	4.5	**2.0**	**7.0**	13.8	0.0	**8.0**	12.0	4.0	
Married or de facto	**72.9**	73.1	72.7	**54.0**	**75.4**	65.5	85.7	**74.0**	64.0	84.0	
Widowed	**6.3**	3.8	9.1	**18.0**	**8.8**	10.3	7.1	**6.0**	8.0	4.0	
Divorced	**8.3**	3,8	13.6	**24.0**	**7.0**	6.9	7.1	**4.0**	4.0	4.0	
Separated (not divorced)	**2.1**	3.8	0.0	**2.0**	**1.8**	3.4	0.0	**8.0**	12.0	4.0	
**Current employment,** %											<0.001
Full-time	**58.0**	73.1	41.7	**14.0**	**19.3**	17.2	21.4	**12.0**	8.0	16.0	
Part-time	**22.0**	11.5	33.3	**32.0**	**26.3**	27.6	25.0	**22.0**	32.0	12.0	
Unemployed	**2.0**	0.0	4.2	**4.0**	**1.8**	0.0	3.6	**0.0**	0.0	0.0	
Retired	**6.0**	7.7	4.2	**32.0**	**47.4**	51.7	42.9	**62.0**	52.0	72.0	
Student	**2.0**	3.8	0.0	**0.0**	**0.0**	0.0	0.0	**0.0**	0.0	0.0	
Home duties	**2.0**	0.0	4.2	**10.0**	**0.0**	0.0	0.0	**2.0**	4.0	0.0	
Pensioner	**8.0**	3.8	12.5	**8.0**	**5.3**	3.4	7.1	**2.0**	4.0	0.0	
**Past/current smoker**, %	**40.0**	34.6	45.8	**22.0**	**38.3**	48.3	33.3	**30.0**	32.0	28.0	0.13
**Osteoporosis**[b], %	**6.0**	3.8	8.3	**42.0**	**5.0**	6.7	3.6	**100.0**	100.0	100.0	<0.001
**BMI** (kg/m^2; mean (s.d.))	**30.6 (5.7)**	29.8 (4.6)	31.5 (6.6)	**23.6 (3.0)**	**32.9 (4.1)**	33.6 (3.7)	32.1 (4.4)	**-**	-	-	<0.001
**SPPB** (# out of 12; median (IQR))	**12.0 (11.0 – 12.0)**	12.0 (11.0 – 12.0)	11.5 (10.5 – 12.0)	**12.0 (11.0 – 12.0)**	**11.0 (10.0-12.0)**	11.0 (10.0-12.0)	11.0 (10.0-12.0)	**-**	-	-	0.004

Abbreviations: BMI, body mass index; SPPB, short physical performance battery.

[a] One-way ANOVA, Kruskal-Wallis test or Pearson's chi-square test was used to determine statistically significant differences between the overall sample of datasets A to D.
[b] Presence of osteoporosis diagnosis was self-reported in Datasets A and D. Dual-energy x-ray absorptiometry scans of the lumbar spine and non-dominant total hip were used to determine presence of osteoporosis in Datasets B and C (defined as T-score ≤ 2.5 at any site).

Supplementary Table 4 Correlations at item level of instruments with hypothesised relationships in shaded cells

		EQ-5D (EQ-5D-3L for Datasets A and B, and EQ-5D-5L for Datasets C and D)				
		Mobility	Self-care	Usual Activities	Pain/ Discomfort	Anxiety/ Depression
**MFES**						
Dressed/undressed	A:	-0.41*	-0.28	-0.30*	-0.35*	-0.37*
	B:	-0.43*	NA[1]	-0.34*	-0.30*	-0.12
	C:	-0.21	-0.05	-0.32*	-0.31*	-0.02
	D:	-0.56*	-0.59*	-0.61*	-0.50*	-0.22
Simple meal	A:	-0.30*	-0.38*	0.00	-0.12	-0.06
	B:	-0.32*	NA	-0.26	-0.31*	-0.07
	C:	-0.42*	0.08	-0.42*	-0.30*	-0.17
	D:	-0.56*	-0.74*	-0.47*	-0.39*	-0.25
Bath/shower	A:	-0.15	-0.32*	-0.17	-0.15	-0.23
	B:	-0.20	NA	-0.30*	-0.24	-0.19
	C:	-0.14	0.09	-0.17	-0.21	-0.05
	D:	-0.58*	-0.42*	-0.79*	-0.60*	-0.24
In/out of chair	A:	-0.32*	-0.28	-0.24	-0.32*	-0.36*
	B:	-0.47*	NA	-0.22	-0.37*	-0.17
	C:	-0.45*	-0.05	-0.53*	-0.40*	-0.16
	D:	-0.72*	-0.58*	-0.62*	-0.51*	-0.22
In/out of bed	A:	-0.41*	-0.25	-0.22	-0.33*	-0.32*
	B:	-0.44*	NA	-0.19	-0.30*	-0.12
	C:	-0.38*	-0.14	-0.45*	-0.29*	-0.02
	D:	-0.72*	-0.59*	-0.62*	-0.51*	-0.11
Answer door/telephone	A:	-0.51*	-0.33*	-0.26	-0.25	-0.20
	B:	-0.57*	NA	-0.07	-0.30*	-0.09
	C:	-0.17	0.07	-0.25	-0.14	0.03
	D:	-0.29*	-0.56*	-0.39*	-0.31*	-0.19
Walk around house	A:	-0.35*	-0.31*	-0.27	-0.29*	-0.39*
	B:	-0.42*	NA	-0.17	-0.29*	-0.11
	C:	-0.26	0.09	-0.32*	-0.29*	-0.04
	D:	-0.56*	-0.59*	-0.57*	-0.44*	-0.11
Reach into cabinet/closet	A:	-0.50*	-0.26	-0.34*	-0.40*	-0.53*
	B:	-0.61*	NA	-0.31*	-0.37*	-0.19
	C:	-0.45*	0.11	-0.39*	-0.27*	-0.05
	D:	-0.55*	-0.57*	-0.68*	-0.58*	-0.17
Light housekeeping	A:	-0.43*	-0.31*	-0.21	-0.25	-0.34*
	B:	-0.56*	NA	-0.30*	-0.31*	-0.17
	C:	-0.18	0.09	-0.19	-0.22	0.06
	D:	-0.34*	-0.55*	-0.64*	-0.50*	-0.29
Simple shopping	A:	-0.30*	-0.30*	-0.09	-0.25	-0.35*
	B:	-0.55*	NA	-0.29*	-0.31*	-0.16
	C:	-0.21	0.09	-0.38*	-0.30*	-0.07
	D:	-0.44*	-0.49*	-0.70*	-0.56*	-0.21
Public transport	A:	-0.54*	-0.29*	-0.36*	-0.35*	-0.44*
	B:	-0.48*	NA	-0.45*	-0.37*	-0.22
	C:	-0.29*	0.07	-0.39*	-0.35*	-0.15
	D:	-0.49*	-0.42*	-0.81*	-0.60*	-0.24
Crossing roads	A:	-0.50*	-0.26	-0.41*	-0.43*	-0.27
	B:	-0.50*	NA	-0.57*	-0.48*	-0.28
	C:	-0.14	-0.07	-0.37*	-0.34*	-0.26*
	D:	-0.68*	-0.39*	-0.69*	-0.60*	-0.11
Light gardening/washing	A:	-0.41*	-0.32*	-0.20	-0.33*	-0.46*

	B:	-0.45*		-0.37*	-0.42*	-0.08
	C:	-0.46*	-0.01	-0.40*	-0.46*	0.10
	D:	-0.48*	-0.43*	-0.79*	-0.61*	-0.22
Using steps at home	A:	-0.55*	-0.22	-0.35*	-0.40*	-0.46*
	B:	-0.56*	NA	-0.45*	-0.44*	-0.08
	C:	-0.30*	-0.01	-0.31*	-0.34*	-0.11
	D:	-0.58*	-0.49*	-0.73*	-0.60*	-0.16
**CDC**						
Self-reported health	A:	0.18	0.12	0.27	0.39*	0.31*
	B:	0.28	NA	0.18	0.31*	0.25
	C:	0.25	0.13	0.11	0.19	0.05
Days physical health not good	A:	0.05	NA	0.23	-0.06	0.25
	B:	0.12	NA	0.11	0.07	0.19
	C:	0.35*	-0.12	0.07	0.18	-0.05
Days mental health not good	A:	0.21	NA	0.17	0.18	0.70*
	B:	0.29*	NA	0.40*	0.09	0.53*
	C:	0.03	-0.15	-0.09	-0.05	0.29*
Limitations in usual activities	A:	0.28	NA	0.48*	0.17	0.18
	B:	0.23	NA	0.73*	0.19	0.18
	C:	0.06	-0.07	-0.10	-0.04	0.00
**WPAI**						
Hours of work missed due to health	D:	0.58*	NA	0.32	0.44	-0.18
Hours of work missed due to other	D:	0.30	NA	0.70*	0.53*	0.12
Hours actually worked	D:	0.07	NA	-0.15	0.00	0.10
Health affect work productivity	D:	0.75*	NA	0.87*	0.75*	0.18
Health affect daily activities	D:	0.63*	0.40*	0.82*	0.73*	0.19

* p<0.05

[1]NA as all responses were the same across sample.

Abbreviations: MFES, Modified Falls Efficacy Scale; CDC, Centre for Disease Control and Prevention; WPAI, Work Productivity and Activity Impairment Questionnaire

Supplementary Table 5 Known group validity of instruments

Groups	N	Mean (s.d.)	Effect Size	N	Mean (s.d.)	Effect Size
**Fear of falling[1]**						
		**EQ-5D-3L**			**CDC Unhealthy days**	
Dataset A						
Lower	33	0.91 (0.11)		33	3.52 (6.42)	
Higher	14	0.74 (0.22)	1.60<br>p<0.01	13	5.92 (8.87)	0.38<br>p=0.27
Dataset B						
Lower	35	0.94 (0.08)		35	5.20 (8.29)	
Higher	13	0.80(0.15)	1.86<br>p<0.01	13	9.73 (12.40)	0.55<br>p=0.21
		**EQ-5D-5L**			**CDC Unhealthy Days**	
Dataset C						
Lower	34	0.95 (0.04)		34	5.50 (8.57)	
Higher	21	0.91 (0.06)	1.12<br>p<0.01	20	4.65 (9.01)	0.10<br>p=0.68
Dataset D					**WPAI (overall work productivity loss)**	
Lower	33	0.95 (0.04)		11	10.91 (27.00)	
Higher	10	0.81 (0.13)	3.73<br>p<0.01	2	15.00 (7.07)	0.15<br>p=0.11
**Frequent distress[2]**						
		**EQ-5D-3L**			**MFES Total Score**	
Dataset A						
Absence	42	0.89 (0.11)		42	9.71 (0.70)	
Presence	7	0.65 (0.27)	2.25<br>p<0.01	7	9.26 (0.99)	0.64<br>p=0.24
Dataset B						
Absence	36	0.93 (0.11)		37	9.75 (0.61)	
Presence	11	0.83 (0.14)	0.98<br>p=0.01	11	9.58 (0.57)	0.28<br>p=0.28
		**EQ-5D-5L**			**MFES Total Score**	
Dataset C						
Absence	50	0.93 (0.06)		47	9.73 (0.43)	
Presence	7	0.93 (0.04)	0.07<br>p=0.83	7	9.87 (0.20)	0.34<br>p=0.36

Groups	N	Mean (s.d.)	Effect Size	N	Mean (s.d.)	Effect Size	N	Mean (s.d.)	Effect Size
**Obesity (BMI ≥30kg/m^2)**									
		**EQ-5D-3L**			**CDC Unhealthy days**			**MFES**	
Dataset A									
Absence	27	0.87 (0.14)		26	3.77 (7.08)		25	9.77 (0.64)	
Presence	23	0.82 (0.20)	0.37<br>p=0.46	23	7.17 (10.04)	0.48<br>p=0.65	22	9.42 (0.97)	0.54<br>p=0.51
		EQ-5D-5L			**CDC Unhealthy days**			**MFES**	
Dataset C									
Absence	17	0.94 (0.05)		16	2.88 (5.55)		16	9.72 (0.36)	
Presence	41	0.93 (0.06)	0.20<br>p=0.77	41	5.78 (9.32)	0.52<br>p=0.17	39	9.75 (0.43)	0.09<br>p=0.77
**Osteoporosis (BMD T-score ≤ -2.5)**									
		**EQ-5D-3L**			**CDC Unhealthy days**			**MFES**	
Dataset B									
Absence	27	0.92 (0.10)		28	5.73 (9.54)		28	9.85 (0.29)	
Presence	21	0.88 (0.14)	0.34<br>p=0.51	20	7.40 (9.97)	0.17<br>p=0.48	21	9.55 (0.83)	1.03<br>p=0.42

[1] Higher fear of falling defined as a MFES total score of <9.8.

[2] Frequent distress defined as a CDC unhealthy days index of ≥14.

Supplemental Table 6 Responsiveness of instruments at the item level

	Dataset		N	% at floor Baseline	% at floor Follow-up	% at ceiling[1] Baseline	% at ceiling[1] Follow-up	Mean change (s.d.)	SRM	p-value
**EQ-5D-3L**										
Mobility	A:	Total	39	0	0	89.7	87.2	0.03 (0.43)	0.06	0.71
	B:	Total	44	0	0	90.9	93.2	-0.02 (0.26)	-0.09	0.56
Self-care	A:	Total	39	0	0	100	100	0 (0.00)	-	-
	B:	Total	44	0	0	100	97.7	0.02 (0.15)	0.15	0.32
Usual Activities	A:	Total	39	0	0	92.3	89.7	0.03 (0.36)	0.07	0.66
	B:	Total	44	0	0	86.4	93.2	-0.07 (0.33)	-0.20	0.18
Pain/Discomfort	A:	Total	39	2.6	5.1	46.2	53.8	-0.05 (0.56)	-0.09	0.57
	B:	Total	43	0	2.3	62.8	58.1	0.07 (0.46)	0.15	0.32
Anxiety/Depression	A:	Total	39	2.6	5.1	74.4	74.4	0.03 (0.36)	0.07	0.66
	B:	Total	43	0	0	69.8	65.1	0.05 (0.43)	0.11	0.48
**EQ-5D-5L**										
Mobility	C:	EX	24	0	0	66.7	83.3	-0.21 (0.66)	-0.32	0.13
		CON	24	0	0	91.7	83.3	0.13 (0.68)	0.18	0.41
	D:	EX	23	0	4.3	56.5	52.5	0.13 (0.76)	0.17	0.38
		CON	20	0	0	80.0	65.0	0.05 (0.60)	0.08	0.71
Self-care	C:	EX	24	0	0	95.8	100	-0.04 (0.20)	-0.20	0.32
		CON	24	0	0	95.8	100	-0.04 (0.20)	-0.20	0.32
	D:	EX	23	0	0	95.7	95.7	0.09 (0.42)	0.21	0.32
		CON	20	0	0	90.0	90.0	-0.05 (0.39)	-0.13	0.56
Usual Activities	C:	EX	24	0	0	87.5	95.8	-0.13 (0.45)	-0.28	0.18
		CON	24	0	0	95.8	83.3	0.13 (0.68)	0.18	0.41
	D:	EX	23	0	4.3	56.5	56.5	0.09 (1.00)	0.09	0.66
		CON	20	0	0	65.0	70.0	-0.10 (0.55)	-0.18	0.41
Pain/Discomfort	C:	EX	24	0	0	4.2	20.8	-0.13 (0.61)	-0.20	0.32
		CON	24	0	0	37.5	25.0	0.08 (0.78)	0.11	0.59
	D:	EX	23	0	0	26.1	21.7	0.09 (0.67)	0.13	0.53
		CON	20	0	0	25.0	25.0	0.10 (0.72)	0.14	0.53
Anxiety/Depression	C:	EX	24	0	0	54.2	70.8	-0.17 (0.70)	-0.24	0.25
		CON	24	0	0	58.3	58.3	0.04 (0.81)	0.05	0.76
	D:	EX	23	0	0	65.2	69.6	-0.04 (0.37)	-0.12	0.56
		CON	20	0	0	60.0	60.0	0.05 (0.51)	0.10	0.66
**MFES**										
Dressed/undressed	A:	Total	39	0	0	74.4	76.9	0.14 (0.91)	0.15	0.36
	B:	Total	44	0	0	86.4	86.4	-0.11 (0.79)	-0.14	0.40
	C:	EX	24	0	0	58.3	58.3	0.43 (1.35)	0.32	0.20

		CON	24	0	0	83.3	83.3	0.04 (0.74)	0.06	0.99
	D:	EX	20	0	0	95.0	95.0	0.00 (0.00)	NA	0.99
		CON	17	0	0	82.4	88.2	0.12 (0.33)	0.35	0.16
Simple meal	A:	Total	39	0	0	84.6	82.1	0.00 (0.13)	0.00	0.99
	B:	Total	44	0	0	90.9	95.5	0.01 (0.59)	0.02	0.60
	C:	EX	24	0	0	75.0	75.0	-0.02 (0.38)	-0.05	0.76
		CON	24	0	0	91.7	95.8	0.08 (0.43)	0.19	0.41
	D:	EX	20	0	0	100	100	0.00 (0.00)	NA	0.99
		CON	17	0	0	88.2	88.2	-0.12 (0.78)	-0.15	0.66
Bath/shower	A:	Total	39	0	0	84.6	84.6	0.05 (0.26)	0.20	0.23
	B:	Total	44	0	0	81.8	88.6	-0.01 (0.71)	-0.02	0.88
	C:	EX	24	0	0	70.8	58.3	-0.22 (0.61)	-0.36	0.09
		CON	24	0	0	87.5	79.2	-0.15 (0.38)	-0.39	0.07
	D:	EX	20	0	0	80.0	100	0.60 (1.39)	0.43	0.07
		CON	17	0	0	82.4	88.2	0.06 (0.43)	0.14	0.56
In/out of chair	A:	Total	39	0	0	79.5	74.4	-0.09 (0.83)	-0.11	0.50
	B:	Total	44	0	0	86.4	88.6	0.00 (0.66)	0.00	0.99
	C:	EX	24	0	0	70.8	62.5	-0.13 (0.94)	-0.13	0.54
		CON	24	0	0	79.2	79.2	-0.04 (0.49)	-0.08	0.77
	D:	EX	20	0	0	85.0	80.0	-0.15 (0.49)	-0.31	0.18
		CON	16	0	0	93.8	87.5	-0.19 (0.54)	-0.34	0.18
In/out of bed	A:	Total	39	0	0	79.5	74.4	-0.07 (0.81)	-0.09	0.60
	B:	Total	44	0	0	84.1	88.6	-0.02 (0.61)	-0.04	0.94
	C:	EX	24	0	0	70.8	66.7	-0.08 (0.40)	-0.21	0.27
		CON	24	0	0	87.5	83.3	0.03 (0.28)	0.09	0.75
	D:	EX	20	0	0	90.0	100	0.15 (0.49)	0.31	0.18
		CON	17	0	0	88.2	82.4	-0.18 (0.53)	-0.33	0.18
Answer door/telephone	A:	Total	39	0	0	82.1	82.1	0.04 (0.76)	0.05	0.75
	B:	Total	42	0	0	90.5	100	0.07 (0.24)	0.30	0.06
	C:	EX	24	0	0	75.0	75.0	-0.05 (0.41)	-0.11	0.58
		CON	24	0	0	95.8	95.8	0.01 (0.11)	0.11	0.66
	D:	EX	20	0	0	100	100	0.00 (0.00)	NA	0.99
		CON	17	0	0	94.1	94.1	0.00 (0.00)	NA	0.99
Walk around house	A:	Total	39	0	0	82.1	84.6	0.05 (0.26)	0.20	0.23
	B:	Total	44	0	0	84.1	90.9	0.02 (0.61)	0.04	0.52
	C:	EX	24	0	0	70.8	75.0	0.02 (0.54)	0.04	0.94
		CON	24	0	0	87.5	87.5	0.07 (0.43)	0.16	0.42
	D:	EX	20	0	0	95.0	100	0.15 (0.67)	0.22	0.31
		CON	16	0	0	87.5	87.5	0.00 (0.00)	NA	0.99
Reach into cabinet/closet	A:	Total	39	0	0	79.5	82.1	0.09 (0.45)	0.20	0.22
	B:	Total	43	0	0	86.0	81.4	-0.15 (0.98)	-0.15	0.29
	C:	EX	24	0	0	62.5	58.3	-0.03 (0.77)	-0.04	0.98

		CON	24	0	0	83.3	75.0	0.01 (0.36)	0.02	0.93
	D:	EX	20	0	0	90.0	85.0	0.00 (0.65)	0.00	0.99
		CON	16	0	0	87.5	81.3	0.13 (0.81)	0.16	0.66
Light housekeeping	A:	Total	39	0	0	84.6	84.6	0.01 (0.15)	0.03	0.83
	B:	Total	44	0	0	81.8	90.9	0.01 (0.50)	0.02	0.86
	C:	EX	24	0	0	75.0	70.8	-0.04 (0.42)	-0.10	0.69
		CON	24	0	0	87.5	87.5	0.06 (0.31)	0.20	0.41
	D:	EX	20	0	0	95.0	90.0	-0.05 (0.69)	-0.07	0.79
		CON	17	0	0	82.4	82.4	-0.12 (0.49)	-0.24	0.32
Simple shopping	A:	Total	39	0	0	82.1	79.5	-0.05 (0.53)	-0.10	0.55
	B:	Total	44	0	0	81.8	88.6	0.00 (0.52)	0.00	0.93
	C:	EX	24	0	0	70.8	70.8	-0.02 (0.46)	-0.05	0.84
		CON	24	0	0	87.5	87.5	0.01 (0.33)	0.03	0.89
	D:	EX	20	0	0	90.0	95.0	0.50 (2.06)	0.24	0.29
		CON	17	0	0	82.4	82.4	-0.35 (1.06)	-0.33	0.18
Public transport	A:	Total	39	0	0	79.5	79.5	-0.03 (0.61)	-0.04	0.79
	B:	Total	44	0	0	72.7	81.8	0.25 (0.96)	0.26	0.09
	C:	EX	23	0	0	52.5	60.9	0.07 (0.96)	0.07	0.69
		CON	22	0	0	90.9	68.2	-0.22 (0.90)	-0.24	0.31
	D:	EX	19	5.3	0	84.2	84.2	0.47 (1.54)	0.31	0.22
		CON	16	0	0	68.8	75.0	0.13 (0.72)	0.17	0.48
Crossing roads	A:	Total	39	0	0	74.4	76.9	0.09 (1.05)	0.09	0.60
	B:	Total	43	0	0	74.4	76.7	0.06 (1.17)	0.05	0.66
	C:	EX	24	0	0	54.2	54.2	-0.03 (0.97)	-0.03	0.82
		CON	24	0	0	66.7	50.0	-0.34 (1.06)	-0.32	0.16
	D:	EX	20	0	0	80.0	85.0	0.40 (1.43)	0.28	0.20
		CON	18	0	0	72.2	83.3	0.11 (0.47)	0.24	0.32
Light gardening/washing	A:	Total	39	0	0	82.1	82.1	-0.04 (0.60)	-0.07	0.65
	B:	Total	44	0	0	77.3	84.1	0.15 (0.99)	0.15	0.44
	C:	EX	24	0	0	58.3	66.7	-0.05 (0.65)	-0.08	0.86
		CON	24	0	0	66.7	75.0	0.39 (1.90)	0.21	0.58
	D:	EX	20	0	0	85.0	90.0	0.55 (2.04)	0.27	0.18
		CON	17	0	0	76.5	70.6	-0.24 (0.66)	-0.35	0.16
Using steps at home	A:	Total	39	0	0	71.8	82.1	0.35 (1.22)	0.29	0.08
	B:	Total	43	0	0	76.7	81.4	0.14 (1.11)	0.13	0.72
	C:	EX	24	0	0	54.2	58.3	0.28 (1.37)	0.20	0.51
		CON	24	0	0	75.0	75.0	-0.04 (0.96)	-0.04	0.96
	D:	EX	17	5.9	0	88.2	88.2	0.53 (2.29)	0.23	0.41
		CON	16	0	0	75.0	81.3	-0.25 (0.86)	-0.29	0.26
**CDC**										
Self-reported health	A:	Total	39	0	0	15.4	20.5	-0.13 (0.57)	-0.22	0.17
	B:	Total	43	0	2.3	11.6	11.6	0.00 (0.58)	0.00	0.99

	C:	EX	24	0	0	8.3	12.5	-0.29 (0.75)	-0.39	0.07
		CON	23	0	4.3	13.0	26.1	-0.13 (1.06)	-0.12	0.17
Physically unhealthy days	A:	Total	39	0	5.1	61.5	38.5	4.22 (7.48)	0.56	**0.01***
	B:	Total	43	7.0	4.7	65.1	53.5	0.65 (6.54)	0.10	0.48
	C:	EX	24	0	0	75.0	54.2	2.08 (6.15)	0.34	0.14
		CON	23	4.3	4.3	73.9	39.1	2.78 (8.49)	0.33	0.08
Mentally unhealthy days	A:	Total	39	2.6	7.7	61.5	59.0	1.36 (6.30)	0.22	0.19
	B:	Total	41	2.4	4.9	61.0	58.5	0.89 (5.92)	0.15	0.49
	C:	EX	24	0	0	70.8	62.5	0.33 (3.73)	0.09	0.72
		CON	22	0	0	59.1	68.2	-1.14 (8.65)	-0.13	0.33
Activity limitation	A:	Total	39	7.7	2.6	79.5	71.8	3.05 (7.66)	0.40	**0.02***
	B:	Total	42	2.4	2.4	78.6	69.0	0.69 (4.13)	0.17	0.09
	C:	EX	23	0	0	95.7	82.6	0.57 (3.55)	0.16	0.34
		CON	23	0	4.3	87.0	56.5	2.17 (9.66)	0.23	0.13
**WPAI**										
Hours of work missed due to health	D:	EX	8	0	0	100	100	0.00 (0.00)	-	0.99
		CON	5	0	0	80.0	100	-1.60 (3.58)	-0.45	0.32
Hours of work missed due to other	D:	EX	8	0	0	87.5	100	-0.75 (2.12)	-0.35	0.32
		CON	4	0	0	100	75.0	9.50 (19.00)	0.50	0.32
Hours actually worked	D:	EX	8	0	0	12.5	0	4.44 (14.94)	0.30	0.36
		CON	5	0	0	0	20.0	-4.00 (19.04)	-0.21	0.47
Health problems affect productivity	D:	EX	8	0	0	62.5	62.5	-0.38 (3.34)	-0.11	0.72
		CON	5	0	0	80.0	100	-0.80 (1.79)	-0.45	0.32
Health problems affect daily activities	D:	EX	23	8.7	4.3	30.4	56.5	-0.65 (3.46)	-0.19	0.31
		CON	21	0	0	52.4	61.9	0.10 (2.00)	0.05	0.87

[1]Ceiling effects reported in this table are the highest possible scores reflecting better quality of life or more favourable outcomes.
NA results were due to similar responses across the sample.
* p<0.01

Supplementary Figure 1 Bland-Altman plots of differences for scores between PROMs in Datasets B (n=50), C (n=60) and D (n=50)

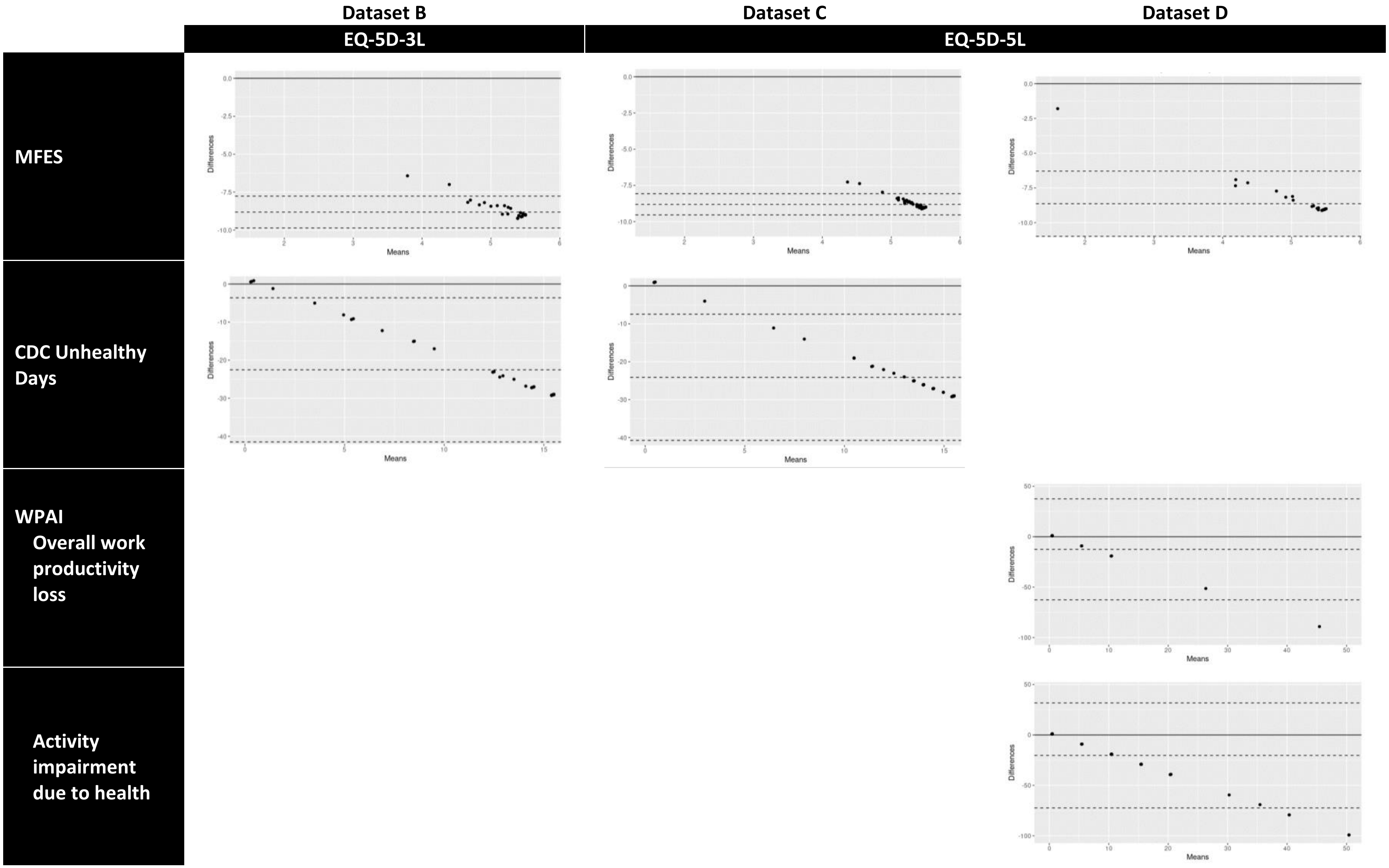

Supplement: Supplemental Material - Exploring the Validity of Measures of Health-Related Quality of Life in Older Adults at Increased Risk of Falls And/Or Fractures in Exercise Clinical Trials [file sj-pdf-1-jag-10.1177_07334648251316633.pdf]
